# Brain Tumor Diagnostics and Therapeutics with Superparamagnetic Ferrite Nanoparticles

**DOI:** 10.1155/2017/6387217

**Published:** 2017-12-11

**Authors:** Fahmeed Hyder, S. Manjura Hoque

**Affiliations:** ^1^Magnetic Resonance Research Center, Yale University, New Haven, CT, USA; ^2^Department of Biomedical Engineering, Yale University, New Haven, CT, USA; ^3^Department of Radiology and Biomedical Imaging, Yale University, New Haven, CT, USA; ^4^Materials Science Division, Bangladesh Atomic Energy Commission, Dhaka, Bangladesh

## Abstract

Ferrite nanoparticles (F-NPs) can transform* both* cancer diagnostics and therapeutics. Superparamagnetic F-NPs exhibit high magnetic moment and susceptibility such that in presence of a static magnetic field transverse relaxation rate of water protons for MRI contrast is augmented to locate F-NPs (i.e., diagnostics) and exposed to an alternating magnetic field local temperature is increased to induce tissue necrosis (i.e., thermotherapy). F-NPs are modified by chemical synthesis of mixed spinel ferrites as well as their size, shape, and coating. Purposely designed drug-containing nanoparticles (D-NPs) can slowly deliver drugs (i.e., chemotherapy). Convection-enhanced delivery (CED) of D-NPs with MRI guidance improves glioblastoma multiforme (GBM) treatment. MRI monitors the location of chemotherapy when D-NPs and F-NPs are coadministered with CED. However superparamagnetic field gradients produced by F-NPs complicate MRI readouts (spatial distortions) and MRS (extensive line broadening). Since extracellular pH (pH_e_) is a cancer hallmark, pH_e_ imaging is needed to screen cancer treatments. Biosensor imaging of redundant deviation in shifts (BIRDS) extrapolates pH_e_ from paramagnetically shifted signals and the pH_e_ accuracy remains unaffected by F-NPs. Hence effect of* both* chemotherapy and thermotherapy can be monitored (by BIRDS), whereas location of F-NPs is revealed (by MRI). Smarter tethering of nanoparticles and agents will impact GBM theranostics.

## 1. Introduction

The prognosis for patients with brain tumors remains poor despite surgical advances [1]. Thousands of intracranial malignancies are reported in United States each year, and, moreover, the incidence rates worldwide are rising faster [2]. The survival rate for patients with glioblastoma multiforme (GBM)—the most common malignant glioma in adults—is a little more than a year [3]. Although surgeries and survival rate improve, most GBMs remain difficult to treat [1]. Therefore novel approaches are needed for improved management of patients with malignant brain tumors, in terms of early diagnosis, tracking therapeutic response, and, of course, improved therapies.

### 1.1. Bypassing the Blood-Brain Barrier to Treat Brain Tumors

Most GBM patients undergoing chemotherapy usually receive drugs systemically. However drugs injected into the body in this manner do not reach the tumor cells in pharmacologically relevant levels [4], which in part could be the reason for tumor recurrence while receiving chemotherapy [5]. An impediment to management of patients with malignant brain tumors is that the blood-brain barrier (BBB) obstructs efficient drug delivery for a vast majority of small- and large-molecule drugs [6]. Compared to vasculature in normal tissue, GBM tumors consist of abnormal vasculature comprised of proliferative, leaky, and unorganized blood vessels with necrotic cores [7]. Various approaches are being utilized to bypass the BBB, for example, implantation of biodegradable wafers [8]. However the most promising methods are based on bioengineered nanomaterials [9], in part, because their sizes are biologically relevant to cells, viruses, proteins, and genes.

**Figure 1:**
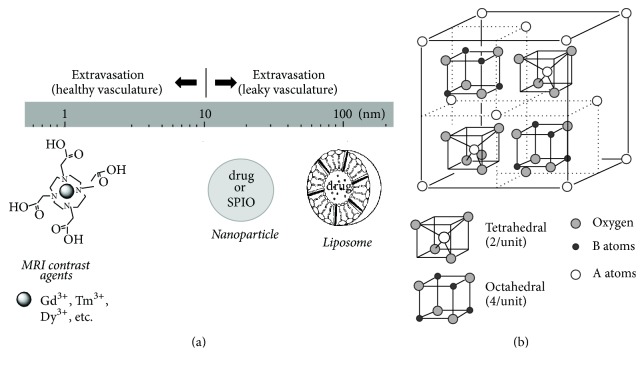
Nanomaterials and MRI contrast agents for cancer theranostics. (a) Size scaling of nanomaterials (e.g., liposomes, nanoparticles) and MRI contrast agents in relation to their extravasation when injected systemically. It is generally believed that in cancer the vasculature is more leaky [7]. Drug and/or SPIO can be encapsulated into nanomaterials. (b) Spinel ferrite structure showing tetrahedral and octahedral sites. There are 64 tetrahedral and 32 octahedral positions that are available for cations in one ferrite unit cell. However 12.5% and 75% of the tetrahedral and octahedral sites, respectively, are occupied by cations. See [14] for details.

A method for drug delivery across the BBB—approved in 1996 by the Federal Drug Agency (FDA)—is to insert drug-eluting materials into the brain, for example, either by direct implantation or by specific molecular targeting [4]. If sufficiently biocompatible nanomaterials are used to load the drugs, then the drug can be slowly released for treating diseases, for example, from neurodegeneration [11] to neurooncology [12]. This method is believed to provide the highest drug concentrations within a specific region of interest (ROI) that are most in need of the treatment, and thus, reduce many systemic side effects. The first nanomedicines were based on liposomes, and today there are several liposomal formulations for clinical therapy [13]. Liposomes are vesicles composed of lipid bilayers with an aqueous inner core, which is ideal for hydrophilic drugs. Nanoparticles are ideal for encapsulating hydrophobic drugs and their structure can be varied according to the design, synthesis, composition, and functionalization. Because many small- and large-molecule chemotherapy drugs are hydrophobic [6], drug-containing nanoparticles (D-NPs) will have significance in treatment of GBM patients [4]. By controlling the size of D-NPs, they maintain long retention times and they are not rapidly eliminated via the reticuloendothelial system (Figure 1(a)). When this feature is combined with the slow delivery of drugs in an ROI, the therapeutic effect can be quite significant [12].

It is believed that most treatments for malignant brain tumor fail because the cancer cells are infiltrative and invade beyond the site of origin [1], BBB blocks intravenous drugs from adequate distribution to regions of cell infiltration [6], and systemic toxicity significantly reduces effectiveness of current therapies [5]. These reasons have been the motivation for extensive developments of improved drug delivery methods. A promising strategy is bypassing the BBB and infusing drugs directly into the tumor with intracranial catheters using a method called convection-enhanced delivery (CED) [23]. Although clinical studies show that CED is safe [24], most CED trials for GBM fail for two reasons: while small- or large-molecule drugs penetrate tissue, they disappear almost as soon as CED infusion stops [25]; infused drugs cannot be precisely delivered to tumor sites without intraprocedural visualization during the CED method itself (i.e., inability to track the location of chemotherapy) [4]. New technologies for D-NPs can meet these challenges. While D-NPs can be engineered for targeted delivery and controlled over long-term for slow drug release [12], loading drugs into nanocarriers like liposomes and nanoparticles (Figure 1(a)) can protect drugs from rapid clearance [26]. Thus use of D-NPs with CED shows some promise for GBM therapies [4].

### 1.2. Magnetic Nanoparticles in Treatment of Cancer

Magnetic nanoparticles (M-NPs) can be manipulated by external magnetic fields to provide a range of biomedical applications, that is, from providing novel cancer therapies to generating contrast for magnetic resonance imaging (MRI) [27]. M-NPs consist of the magnetic (e.g., inner core consisting of specific metal oxides) and chemical (i.e., outer core designed for functionality) components. M-NPs less than 30 nm in diameter become superparamagnetic to exhibit high magnetic moment and susceptibility—behavior that appears in both ferromagnetic or ferrimagnetic materials—but superparamagnetic properties are also controlled by how they are composed. Ferromagnetism occurs in several metallic elements because the magnetic moments align parallel to produce strong permanent magnets. Ferrimagnetism is similar to ferromagnetism (e.g., spontaneous magnetization, Curie temperatures, and magnetic hysteresis), but this biocompatible family is based on superparamagnetic iron oxide nanoparticles (SPIO-NPs) and occurs in magnetite (Fe_3_O_4_) or its oxidized form, maghemite (*γ*-Fe_2_O_3_). Net magnetic moment arises when these nanomaterials are exposed to external magnetic fields, static or alternating.

SPIO-NPs combined with one or more additional metallic elements (e.g., Fe, Ni, Co, and Zn) are called ferrites and like M-NPs they have applications across a range of disciplines, from biomedical to industrial [14]. F-NPs are metal oxides with spinel structure with an AB_2_O_4_ formula, where A and B are cations located tetrahedrally (A atom; smaller and two per unit) and octahedrally (B atom; larger and four per unit) coordinated to oxygen atoms, respectively (Figure 1(b)). Recent studies show that F-NPs have novel applications in different areas of biomedical engineering [28, 29], for example, drug delivery [30], MRI contrast generation [31], and hyperthermia treatments [32]. One of the ways targeted cancer cells are killed is by heat induced by F-NPs on its immediate environment, which is achieved by exposing them to an alternating magnetic field (AMF) [14].

### 1.3. Magnetic Resonance Methods in Treatment of Cancer

**Figure 2:**
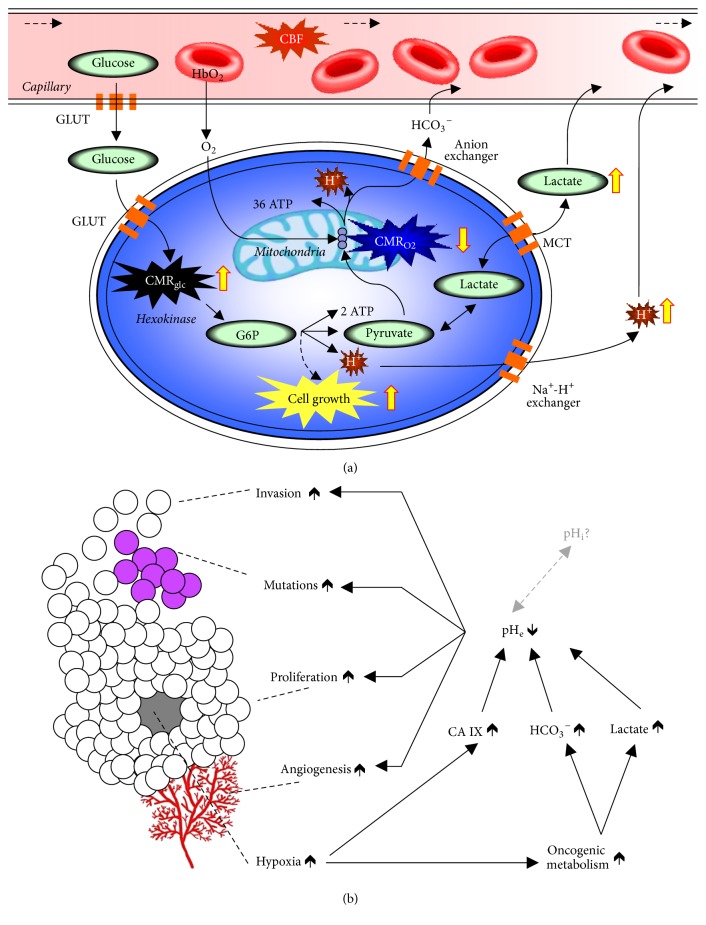
Relationship between tumor metabolism and tumor biology. (a) The tumor microenvironment, compared to the normal neuropil, is characterized by aerobic glycolysis and endothelial dysfunction [15]. CBF, cerebral blood flow; GLUT, glucose transporter; HbO_2_, oxyhemoglobin; G6P, glucose-6-phosphate; H^+^, hydrogen ions; CMR_glc_, glycolysis; CMR_O2_, oxidative phosphorylation; MCT, monocarboxylic acid transporters. To maintain intracellular pH homeostasis acidic constituents are extruded out to acidify the extracellular space. (b) Tumor biology is intricately linked to cancer metabolism. Tumor growth (i.e., invasion, mutation, and proliferation) has been linked to acidic pH_e_ arising from aerobic glycolysis [15]. The altered metabolic pathways, to support cell growth, create byproducts that are actively exported out of tumor cells to help maintain neutral intracellular pH (pH_i_), but this comes at the cost of acidification of the extracellular milieu (pH_e_). These multifaceted processes all decrease pH_e_ while maintaining near normal pH_i_, which lead to tumor invasion, mutations, proliferation, and even angiogenesis.

Tissue contrast with MRI relies on relaxation of water protons (i.e., transverse (*T*_2_^*∗*^ or *T*_2_) and longitudinal (*T*_1_) time constants). Thus contrast depends on varying degrees of intrinsic values of transverse (*R*_2_^*∗*^ = 1/*T*_2_^*∗*^ by gradient-echo or *R*_2_ = 1/*T*_2_ by spin-echo methods) and longitudinal (*R*_1_ = 1/*T*_1_ by inversion recovery or saturation recovery methods) relaxation rates of tissue water protons across tissues. However to generate additional distinction between tissues (e.g., tumor versus normal tissue) MRI contrast agents are used to enhance the relaxation rates, where the ROI darkens and brightens with *R*_2_^*∗*^ (or *R*_2_) and *R*_1_ agents, respectively. Gd^3+^ is a widely used *R*_1_ agent, and it is the most efficient of all paramagnetic lanthanide ions at relaxing water protons because of its seven unpaired electrons. The FDA-approved Gd^3+^-based agents, to reduce systemic toxicity, are complexed with macrocyclics, e.g., DOTA^4−^ (1,4,7,10-tetraazacyclododecane-1,4,7,10-tetraacetic acid) (Figure 1(a)) or variants like DOTMA^4−^ (1,4,7,10-tetramethyl 1,4,7,10-tetraazacyclododecane-1,4,7,10-tetraacetate), DOTP^8−^ (1,4,7,10-tetraazacyclododecane-1,4,7,10-tetrakis methylene phosphonate), and DTPA^5−^ (diethylenetriaminepentaacetic acid). Feridex is an FDA-approved version of SPIO-NPs, which is a strong *R*_2_^*∗*^ (or *R*_2_) agent (Figure 1(a)). While the above-mentioned MRI methods are widely used to measure the tumor location and size, other MRI and MRS methods are used to measure the metabolic dysfunction of cancer cells (Figure 2).

A new MRI contrast called chemical exchange saturation transfer (CEST) is generated when a radio frequency (RF) pulse saturates a pool of exchangeable protons (e.g., amide/amine (-NH_*x*_) or hydroxyl (-OH) protons) to decrease the steady-state proton signal arising from bulk water [33]. The amide proton transfer (APT) version of CEST, generated from proton exchange between protons of water and protons of endogenous mobile proteins and peptides, is enabled by saturation at 3.5 ppm downfield water. Given that such exchangeable protons (assumed to be arising from endogenous mobile proteins and peptides in the cytoplasm) are abundant in tumor tissues compared to healthy tissues, generally 3-4% APT contrast increase is observed in intratumoral region compared to peritumoral region. The APT contrast increase suggests a rise in intracellular pH (pH_i_). While further validation is needed for APT contrast, this has been shown to be responsive to temozolomide treatment in GBM [34]. The amine and amide concentration-independent detection (AACID) is another CEST contrast that combines influences from* both* amine and amide protons in a ratiometric manner such that the need to know the concentration of the exchangeable pool is removed [35]. The AACID contrast, enabled by independent saturations at both 2.75 ppm (amine) and 3.5 ppm (amide) downfield of water, has been validated to pH_i_ measured by ^31^P MRS, which can measure pH_i_ from endogenous inorganic phosphate (P_i_) shift and extracellular pH (pH_e_) from exogenous 3-aminopropyl phosphonate (3-APP) shift, where 3-APP is nontoxic and does not cross the cell membrane [36]. The pH_e_ can also be measured by CEST methods, where the exchangeable proton group resides on the injected agent, which may or may not consist of a paramagnetic cation (i.e., diamagnetic CEST or paramagnetic CEST) [37, 38].

Recently a chemical shift imaging (CSI) based molecular imaging method called biosensor imaging of redundant deviation in shifts (BIRDS) was developed [39, 40], which uses the nonexchangeable protons on agents like TmDOTP^5−^ (i.e., not the effect of the agent on water proton relaxation) as the physiological readout. BIRDS agents demonstrate high signal-to-noise ratio (SNR) and specificity from the paramagnetically shifted proton signals, and this has been used for metabolic imaging (e.g., pH_e_) in a wide range of brain tumors [20, 21, 41].

### 1.4. Metabolic Markers of Cancer

**Figure 3:**
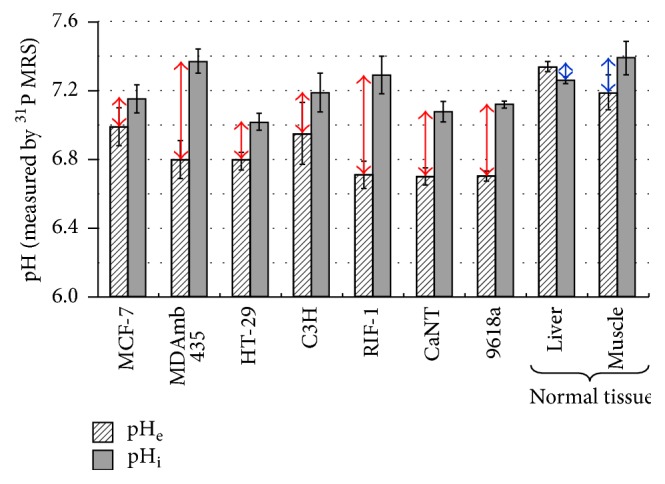
^31^P MRS detection of 3-aminopropyl phosphonate (3-APP) and inorganic phosphate (P_i_) for extracellular pH (pH_e_) and intracellular pH (pH_i_), respectively. The pH_i_-pH_e_ gradient for different types of tumor (red arrows) and normal tissue (blue arrows). From [16] with permission.

Metabolic studies describe the amount of energy needed for cellular building blocks versus cellular functional activity [42]. While these metabolic distinctions are fundamental for quantitative functional brain imaging [42], they are also very pertinent to cancer imaging because it is a disease that reflects out of control cell growth [15]. It is well known that rapidly growing cancer cells have high glycolytic rate (CMR_glc_) in relation to rate of oxidative demand (CMR_O2_) [43]. All gliomas, specifically malignant brain tumors that progress to GBM, demonstrate uncoupling between CMR_glc_ and CMR_O2_ even in presence of sufficient oxygen and thus generate excess lactate and H^+^ in the intracellular space [15]. This process is known as aerobic glycolysis or the Warburg effect [43]. If these acidic constituents are not extruded out appropriately, their presence can radically perturb intracellular function (Figure 2(a)). Thus measuring the acidification of the extracellular milieu is very important for cancer research [15, 44], as extracellular acidosis affects many pathways linked to tumor growth (Figure 2(b)). However tumor cells have alkaline (or near neutral) pH_i_ compared to pH_e_ [16].  Figure 3 shows the pH_i_-pH_e_ gradients measured in a variety of tumors versus normal tissue [16, 45, 46], indicating that pH_e_ is more acidic in tumors whereas pH_i_ in tumors is near neutral to alkaline. Thus measuring both pH_e_ and pH_i_ could be very important for brain cancer research [15]. The superparamagnetic field gradients formed by F-NPs (i.e., >5 mg/kg Fe^3+^) hinder molecular readouts, specifically at high static magnetic field (*B*_*o*_), due to spatial distortions from severe relaxation enhancement for MRI contrast [47] and extensive line broadening reducing MRS specificity/sensitivity [48]. Thus novel imaging methods are needed that are compatible with F-NPs so that* both* their physical location and therapeutic impact could be assessed, because these types of information will greatly benefit subsequent applications to GBM patients.

### 1.5. Outline

This review examines the value of F-NPs to impact* both* diagnostics and therapeutics for GBM treatment in preclinical translational research. The narrative begins with synthesis and characterization of F-NPs, because these procedures affect their magnetic properties and hence their ability to function as MRI contrast agents and provide AMF-induced heat therapy (Section 2). While MRI spots the location of F-NPs, the location of D-NPs is only monitored (by MRI) when D-NPs and F-NPs are coadministered with CED (Section 3). Since F-NPs generate large superparamagnetic fields specifically when delivered by CED, they obscure molecular readouts from* both* MRI (i.e., spatial distortions [47]) and MRS (i.e., extensive line broadening [48]). Given that pH_e_ is a biomarker of cancer progression and treatment, recent developments with a novel pH_e_ mapping method called BIRDS shows that readout capability remains unaffected by presence of F-NPs. In essence these results suggest that effects of* both* impact and location of therapy (i.e., chemotherapy from D-NPs and thermotherapy from F-NPs) can be monitored simultaneously (Section 4). Finally, smarter designs for nanoparticles and agents (i.e., better targeting, reduced toxicity, and higher SNR) in conjunction with the aforementioned bioengineering and bioimaging advances can considerably advance GBM theranostics in the near future (Section 5).

## 2. Synthesis and Characterization of Ferrite Nanoparticles

The effectiveness of M-NPs as mediators for hyperthermia has been studied since 1957 by Gilchrist and coworkers [49] who first conducted the heat induction experiments on M-NPs. Since the early hyperthermia studies with M-NPs, knowledge about heating mechanisms of M-NPs has advanced [50]. The magnetic heating of M-NPs originates from magnetic losses associated with the magnetization-demagnetization cycling, a process known as specific loss power (SLP), which is the ability for nanoparticles to dissipate energy in terms of heat. The SLP depends on eddy current loss, hysteresis loss, and residual loss, where the latter two are more critical for F-NPs.

Eddy current loss is induced by the AMF and thus depends on electrical resistivity. Magnetic heating is initiated by hysteresis loss and residual loss during AMF. Hysteresis loss is due to reversing the magnetization in the alternating current of the magnetic field, which is proportional to the area of the hysteresis loop. The residual loss is due to relaxation effects. When the alternating field is applied to M-NPs, their magnetic moments rotate following the magnetic field with an effective relaxation time (*τ*):

(1a) $$\begin{array}{c} \frac{1}{\tau }=\frac{1}{\tau_{N}}+\frac{1}{\tau_{B}}, \end{array}$$

where *τ*_*N*_ is the Néel relaxation time:

(1b) $$\begin{array}{c} \tau_{N}=\tau_{o}e^{KV/kT} \end{array}$$

and *τ*_*B*_ is the Brownian relaxation time:

(1c) $$\begin{array}{c} \tau_{B}=\frac{3\eta V_{H}}{kT} \end{array}$$

and *τ*_*o*_ is the time constant with the value of 10^−9^ s, *k* is the Boltzmann constant, *T* is the temperature, *η* is the viscosity of the carrier fluid, *K* is the anisotropy constant of the nanoparticle, *V* is the volume of the nanoparticle, and *V*_*H*_ is the hydrodynamic volume of the nanoparticle. These indicate that the anisotropy, hydrodynamic volume, and physical size of the nanoparticle all greatly influence the effective relaxation time. Since the magnetic (i.e., magnetic moment and susceptibility) and physical (i.e., size, shape, and distributions) properties of the M-NPs dictate their behavior upon AMF induction (see [14] for details), thus SLP has to be measured per nanomaterial:

(2) $$\begin{array}{c} SLP=\frac{C}{m}\frac{\Delta T}{\Delta t}V_{s}, \end{array}$$

where *C* is the sample heat capacity (i.e., the mass weighted mean value of the nanoparticle and water), *m* is mass of the magnetic materials, *V*_*s*_ is the sample volume, and Δ*T*/Δ*t* is evaluated from the initial temperature rise (Δ*T*) over time (Δ*t*). Since the nanoparticle *C* is small (i.e., tiny samples), water heat capacity (4.18 Jg^−1^K^−1^) is considered instead for SLP estimation.

Since F-NPs are superparamagnetic (i.e., high magnetic moment and susceptibility), they provide strong enhancement of the observed transverse relaxation rate (*R*_2_):

(3a) $$\begin{array}{c} R_{2}=R_{2}^{0}+r_{2}C_{NP}, \end{array}$$

where *r*_2_ is the contrast agent's relaxivity, *R*_2_^0^ is the intrinsic relaxation rate, and *C*_NP_ is the nanoparticle concentration. Advanced MRI relaxation theories [51, 52] show that *r*_2_ increases in the motional averaging regime (also known as the other sphere model), *r*_2_ reaches maximum in the static dephasing regime, and *r*_2_ decreases in the echo-limited regime. The diffusional motion is very fast in motional averaging regime and it follows that

(3b) $$\begin{array}{c} R_{2}=\frac{16}{45}f\tau_{D}{\left(\;\Delta \omega \;\right)}^{2}, \end{array}$$

where *f* is the volume fraction occupied by the nanoparticles in the suspension, Δ*ω* (=*γμ*_*o*_*M*_*v*_/3) is the angular frequency shift experienced by a proton at the equator of the nanoparticle, *γ* is the proton gyromagnetic ratio, *M*_*v*_ is the volume saturation magnetization, *μ*_*o*_ is the magnetic permeability of vacuum, and *τ*_*D*_ (=*d*_NP_^2^/4*D*;* D* is the water diffusion constant and *d*_NP_ is the particle diameter) is the translational diffusion time of protons in magnetic field inhomogeneity created by nanoparticles. When water protons surround a small space compared to the outer shell (or hydrodynamic diameter) around the nanoparticle, the static dephasing regime is invoked and it follows that

(3c) $$\begin{array}{c} R_{2}=\frac{2\pi }{3\sqrt{3}}f\Delta \omega . \end{array}$$

For larger nanoparticles or agglomeration of smaller nanoparticles, *R*_2_ is governed by the echo-limited regime where neither the motional averaging nor static dephasing regimes are effective. Overall, the factors that mediate *R*_2_ are

(3d) $$\begin{array}{c} R_{2}=\frac{A}{d_{NP}}\frac{1}{D}\gamma^{2}\mu^{2}C_{NP}J\left(\;\omega ,\tau_{D}\;\right), \end{array}$$

where *A* is a constant, *µ* is the magnetic moment of the nanoparticle, and *J*(*ω*, *τ*_*D*_) is the spectral density function. Since *R*_2_ decreases directly with *µ* and inversely with *d*_NP_, nanoparticles with higher moment and smaller size would reduce the amount of particles necessary to obtain efficient MRI contrast enhancement.

Physical properties of F-NPs can be tailored by controlling their size, morphology, and composition, whereas surface chemistry could be achieved by varying pH of solution, ionic strength, capping agent, reaction temperature, and ambient atmosphere [30–32]. Significant variations in anisotropy can be introduced by changing the shape of the nanoparticles [53], but this must happen in conjunction with the physicochemical characteristics of the nanoparticles that exert enhanced permeability and retention within the ROI to avoid nonspecific interactions [54]. F-NPs are not colloidally stable in aqueous media near neutral pH because the surface of the metal oxide is not electrostatically charged. Thus to provide electrostatic or steric repulsion, the nanoparticles can be stabilized by biocompatible ligands, e.g., polyethylene glycol (PEG), poly(lactic-co-glycolic acid) (PLGA), dextran, or chitosan.

Lee and coworkers [50] developed highly anisotropic F-NPs with compositions of CoFe_2_O_4_@MnFe_2_O_4_, CoFe_2_O_4_@Fe_3_O_4_, MnFe_2_O_4_@CoFe_2_O_4_, and Fe_3_O_4_@CoFe_2_O_4_, where SLP was enhanced by one order of magnitude by coating compared to the bare components. They also compared the therapeutic effect of their anisotropic F-NPs and doxorubicin versus the isotropic Feridex and doxorubicin on cancer cells. Feridex is an FDA-approved version of SPIO-NPs, whereas doxorubicin is an anticancer chemotherapeutic drug. The F-NPs provided only thermotherapy upon AMF induction, whereas Feridex could provide AMF-induced thermotherapy and doxorubicin could provide continuous chemotherapy. The thermotherapy of F-NPs was shown to be more effective than Feridex (thermotherapy) and doxorubicin (chemotherapy) together. While this study showed that anisotropic F-NPs are far more effective in heat-induced mortality of cancer cells compared to isotropic SPIO-NPs, mechanisms relating anisotropy to SLP are still under debate.

Nándori and Rácz [55] studied hyperthermia with anisotropic M-NPs focusing on SLP under circularly polarized field. They found that below a critical anisotropy level the SLP remained unaltered, while above that critical limit SLP diminished with increasing anisotropy. Fortin and coworkers [56] studied a variety of different anisotropic maghemite and cobalt ferrite dispersed in aqueous suspension by electrostatic stabilization. They reported that the most important factors for heat are particle size, solvent viscosity, magnetic anisotropy, and the AMF frequency and amplitude. Prado and coworkers [57] demonstrated that enhancing anisotropy of maghemite by surface coordination enhanced the magnetic properties; that is, anisotropy increased from 26 to 65 kJ/m^3^, the blocking temperature (i.e., below this temperature magnetization is lost) increased from 11 to 30 K, and coercivity (i.e., resistance of a material to magnetization changes) increased from 62 to 839 Oe.

Poperechny and coworkers [58] performed experiments with single domain nanoparticles to show that with a uniaxial anisotropy low AMF frequency magnetic hyperthermia can be achieved. Habib and coworkers [59] showed that while the heating rate of Fe-Co nanoparticles increased with nanoparticle size, the heating rate increased/decreased with low/high anisotropy, that is, in the range of 5–25 kJ/m^3^ and 50–400 kJ/m^3^, respectively. These suggest that larger nanoparticle size with lower anisotropy may be more favorable for hyperthermia, but at the same time access of larger nanoparticles can provide delivery challenges. Given that Tackett and coworkers [60] showed a significant magnetocrystalline anisotropy difference between Fe_3_O_4_ and CoFe_2_O_4_ nanoparticles (i.e., 14 and 380 kJ/m^3^), Hoque and coworkers [10] hypothesized that the Fe-Co mixed spinel system will allow for effective tuning of the anisotropy.

Hoque and coworkers [10] synthesized Fe-Co mixed spinel ferrites by chemical coprecipitation (i.e., Fe_*x*_Co_1−*x*_Fe_2_O_4_, where *x* = 0.8, 0.6, 0.4, 0.2) using NH_4_OH or NaOH, which required molar ratio of [M^2+^]/[Fe^3+^] at 1 : 2 where M^2+^ is the salt of divalent Fe and Co dissolved in distilled water. To avoid Fe^2+^ oxidation, all reactions were carried out in N_2_ at normal room temperature. Highly concentrated coprecipitating agents were quickly added under basic conditions (pH = 11) and the precipitate was collected through high centrifugation. The magnetic properties were analyzed by superconducting quantum interference device magnetometer at room temperature. Structural characterizations were performed by X-ray diffraction (XRD) and transmission electron microscopy (TEM). The grain size was determined from the width of the 311 peak in the XRD spectrum, whereas the lattice parameter was measured from the *d* values and the peak positions of respective XRD spectra. Mössbauer spectroscopy examined the Fe valence. The blocking temperature was measured by zero-field cooled measurements of bare nanoparticles with an applied field lower than the coercive field. The hydrodynamic diameter was measured by dynamic light scattering. MRI relaxivity was measured at *B*_*o*_ of 11.7 T. AMF-induced heat induction heating was carried out on a solid-state induction power supply with a field of 400 kHz and amplitude of 76 mT. Toxicity profile of coated Fe-Co mixed spinel ferrites was examined on 9L tumor cell cultures.

**Figure 4:**
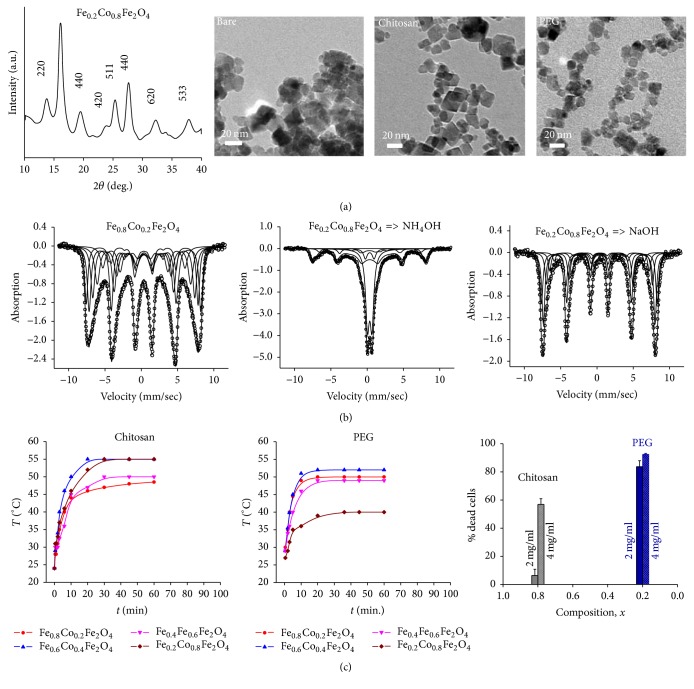
Characterization of Fe_*x*_Co_1−*x*_Fe_2_O_4_ nanoparticles. (a) XRD (left) and TEM (from left to right) data of Fe_0.2_Co_0.8_Fe_2_O_4_ with bare nanoparticles as well as chitosan and PEG-coated nanoparticles, respectively. (b) Mössbauer spectra at room temperature for the compositions Fe_0.8_Co_0.2_Fe_2_O_4_ with NH_4_OH coprecipitating agent (left), Fe_0.2_Co_0.8_Fe_2_O_4_ with NH_4_OH coprecipitating agent (middle), and Fe_0.2_Co_0.8_Fe_2_O_4_ with NaOH coprecipitating agent (right). Slow relaxation is observed for *x* = 0.8 and *x* = 0.2 synthesized by NH_4_OH and NaOH, respectively, as the coprecipitating agent, while other compositions showed mixture of slow/fast relaxation. (c) Heating profiles of Fe-Co mixed spinel ferrites at a concentration of 2 mg/mL of the nanoparticles in water for different compositions coated with chitosan (left) and PEG (middle). The right panel shows the concentration dependence of the nanoparticles in solution for mortality of 9L cells exposed to AMF-induced heating for 30 minutes with chitosan (gray) and PEG (blue) coating. From [10] with permission. See Table 1 for other details [10].

The Fe_*x*_Co_1−*x*_Fe_2_O_4_ characterization data are shown in Figure 4 and Table 1. Although the shapes of the nanoparticles were slightly nonspherical in the bare and coated states, the TEM bright field images of the bare nanoparticles showed some agglomeration, but upon coating the nanoparticles were well dispersed (Figure 4(a)). The average size of the nanoparticle from TEM in the bare state (~7 nm) was different from the XRD data (3–5 nm) (Table 1). The XRD patterns were representative of high crystallinity with well-indexed peaks associated with single-phase ferrites. The grain sizes ranged from 3 to 5 nm. The lattice parameters were slightly larger (~8.5 nm) than the TEM depicted size (~7 nm), whereas the hydrodynamic diameter was about an order of magnitude larger with chitosan or PEG coatings (100–150 nm). The bulk size parameters remained relatively constant with increasing Co content and no significant difference was found with the coprecipitation techniques. However with increasing Co content the blocking temperature and maximum magnetization both increased significantly. The maximum magnetization increased with coating versus bare nanoparticles, and these patterns were also observed for coercivity and MRI relaxivity.

The Mössbauer spectra (Figure 4(b)) demonstrated slow relaxation for Fe_0.8_Co_0.2_Fe_2_O_4_ and Fe_0.2_Co_0.8_Fe_2_O_4_ synthesized by chemical coprecipitation using NH_4_OH and NaOH, respectively, whereas a mixture of slow/fast relaxation was observed for all other compositions. These spectra were best fitted with 4–6 components corresponding to Fe^3+^ situated on the tetrahedral A sites of the spinel structure, while the remaining Fe^2+^ and Fe^3+^ were on the nonequivalent octahedral B sites (see Figure 1(b)). AMF-induced heating for the nanoensembles was quite comparable with either coating except for PEG-coated Fe_0.2_Co_0.8_Fe_2_O_4_, which was twice as less efficient as the others (Figure 4(c)). AMF-induced cell death of 9L tumor cells showed no significant differences with either coating using a dose of 4 mg/mL (Figure 4(c)). This study with Fe-Co mixed spinel ferrites suggests that chitosan and PEG coating of most compositions will have good potential for cancer therapy. While another study by Hoque and coworkers [61] on the Fe-Zn mixed spinel system with regard to chitosan and PEG coatings arrives at a very similar conclusion, other biocompatible coatings for nanoparticles like PLGA [19] and dextran [62] could also be considered for future considerations. However it is crucial to assess the toxicity of any nanoparticle, either bare or in conjunction with its coating [63].

## 3. Monitoring the Location of Chemotherapy from Drug-Containing Nanoparticles

If D-NPs are deposited into a specific ROI, then, designing F-NPs in the same fashion as the D-NPs, the location of chemotherapy delivered by the nanoparticles can also be observed with MRI. CED is a strategy to facilitate targeted delivery of drugs into specific brain ROIs [23]. The insertion of microcatheters directly towards the brain tumor, as guided by MRI, is considered to be a minimally invasive surgical procedure. While CED of various types of drugs has been shown to be clinically safe [24], monitoring the actual convective process or the volume of injected drug has proven to be vital in the optimization of the CED procedure [64].

**Figure 5:**
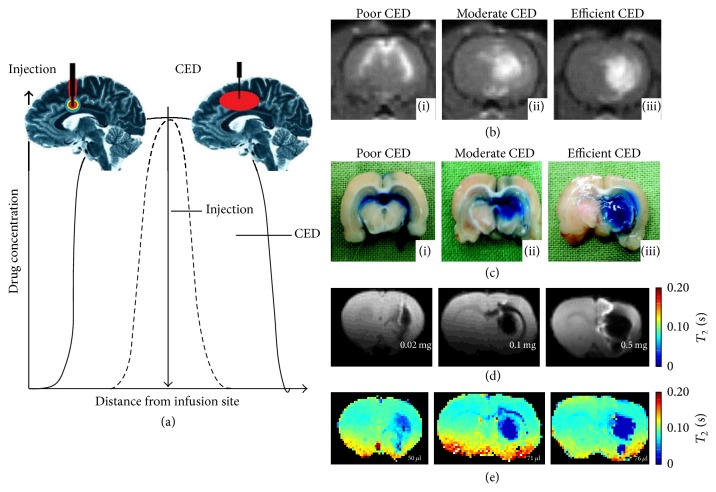
Theoretical and experimental considerations of CED for effective drug delivery. (a) 2D representation of effective convection-enhanced region with intraparenchymal drug injection (small red circle; black dashed line) and CED injected drug (large red oval; black solid line). From [17] with permission. CED in normal rat brain, where the infusate contained GdDTPA^2−^ and Evans blue together, showing examples from three separate rats with different effective convection-enhanced region as depicted by (b) *T*_1_-weighted MRI at *B*_*o*_ of 3.0 T and (c) Evans blue staining for poor, moderate, and efficient CED conditions. From [18] with permission. CED demonstrated with *T*_2_-weighted MRI at *B*_*o*_ of 4.0 T, where the 20 *µ*L infusate contained 0.02 mg, 0.1 mg, and 0.5 mg of SPIO-NPs injected into three normal rats resulting in doses of 1, 5, and 25 mg/kg Fe^3+^, respectively. These respective Fe^3+^ doses were represented as either (d) spin-echo images with an echo time of less than 80 ms and (e) absolute *T*_2_ images. The effective volumes were 50 *µ*L, 71 *µ*L, and 76 *µ*L, respectively, based on either the spin-echo images in (d) or *T*_2_ images in (e). From [19] with permission.

Theoretical considerations [17] suggest that the effective delivery volume by a CED injection is much larger than an intraparenchymal injection (Figure 5(a)). Contrasting CED with diffusion-based methods clarifies the difference. The drug injected directly into the parenchyma usually requires a larger cannula (up to 2 mm, e.g., for microdialysis), and thus this process typically displaces the parenchyma at the tip of the cannula to form a cavity-like bulb from which diffusion of the drug occurs only within that small tissue volume. Hence the primary mechanism of drug spread with intraparenchymal injection is diffusion and the distance is usually a few mm around the cannula tip. The typical CED cannula is much narrower (150–200 *µ*m) and is intricately attached to a finely controlled pump that provides very slow infusion rates (0.2–5.0 *µ*L/min). This allows pressurized extracellular bulk flow in addition to diffusion (i.e., convection plus diffusion) so that the homogenous distribution of materials in the infusate can spread across significant distances (estimated to be up to several cm in the human brain) from the infusion cannula tip. Moreover, typically the infusion cannula extends beyond the outer guide to minimize reflux or backflow along the cannula. When these effective volume advantages of CED (versus intraparenchymal injection methods) are combined with nanoparticles (which provide slow delivery over extended durations), the impact for treatment can be quite significant.

Practical considerations need to accurately image the convective volume of the infusate [64]. Previously Zhou and coworkers [12] conjugated N-(4-[^18^F]fluorobenzyl) propanamido—a positron emission tomography (PET) tracer—to the surface of D-NPs and injected them by CED into rat brain to demonstrate the effective volume of D-NPs by PET. While PET was useful in this diagnostic purpose of the tracking the D-NPs, due to the short half-life of PET isotopes and high cost of PET scans, recent investigations have attempted to measure the effective CED volume by both *T*_1_-weighted [18] and *T*_2_-weighted [19] MRI methods so that continuous monitoring of the infusate volume in relation to the tumor itself can be obtained.

Mardor and coworkers [18] used *T*_1_-weighted MRI in normal rat brain (cannula in striatum) to show differences between poor, moderate, and efficient CED experiments. They determined the effective volume of CED by mixing GdDTPA^2−^ and Evans blue in the infusate prior to infusion, where the latter ex vivo test validated the former in vivo test. High correlation between the GdDTPA^2−^ (Figure 5(b)) and Evans blue (Figure 5(c)) data was observed, suggesting that *T*_1_-weighted MRI can accurately represent the CED volume. In these experiments the CED conditions were purposely varied so that poor, moderate, and efficient CED volumes were achieved. For example, poor CED was characterized by significant backflow along the catheter and into the ventricles with almost no enhancement in the striatum, whereas efficient CED was represented by even spread in the striatum with minimal backflow into the ventricles. Similarly, Strohbehn and coworkers [19] used *T*_2_-weighted MRI in normal rat brain (cannula in striatum) to measure effective volumes for different amounts of D-NPs injected by CED (Figures 5(d) and 5(e)). Using SPIO-NPs (1–25 mg/kg Fe^3+^), they tracked the D-NPs injected with both spin-echo and *T*_2_ images over several weeks. Given that the SNR of *T*_2_-weighted MRI is higher, this method is preferred over the *T*_1_-weighted MRI method.

## 4. Monitoring the Therapeutic Response in Presence of Ferrite Nanoparticles

Recently a ^1^H MRS method called BIRDS was introduced [39, 40, 65] to meet the challenge of providing absolute pH_e_ readout in presence of large superparamagnetic field gradients created by F-NPs, specifically when delivered by CED. It is based on detecting the paramagnetic agent itself (e.g., TmDOTP^5−^), and it combines high molecular specificity/sensitivity in the same 3D-CSI platform, where the pH_e_ readout even at 1 *µ*L resolution provides insights into metabolism of the tumor versus the neighboring healthy/nontumor tissue.

The essence of the method is as follows. While a ^1^H spectrum of the chelating agent (e.g., without the Tm^3+^ ion) shows conventional diamagnetic shifts spread over a narrow range (~5 ppm), a ^1^H spectrum of the complexed agent (e.g., with the Tm^3+^ ion) shows unusual paramagnetic shifts spread much farther apart (>100 ppm). These extremely wide paramagnetically shifted signals also have unusual relaxation properties (i.e., *T*_1_ and *T*_2_ of the paramagnetic protons are in the ms range, instead of hundreds to thousands of ms for typical diamagnetic protons) because the protons are proximal to the unpaired electrons [66]. Because of the extremely short relaxation times of these widely shifted signals, ultrashort excitation RF pulses are used (i.e., *µ*s range), and by using the Shinnar-Le Roux algorithm for the RF pulses, the signals on both sides of water can be simultaneously excited without exceeding FDA safety limits for in vivo MRI/MRS experiments.

These relaxation/shift properties of the probe (e.g., TmDOTP^5−^) enable high-resolution and high speed CSI, and, furthermore, the signals are impervious to poor *B*_*o*_ shim conditions. Because the *T*_2_/*T*_1_ ratio remains high (i.e., ~1 for paramagnetic protons versus ~0.1 for diamagnetic protons), the molecular readout is largely unaffected across different *B*_*o*_. While all of these features of paramagnetic agents combined into one imaging platform for BIRDS are distinctive [20–22, 39–41, 67, 68], there are other studies using these types of agents for bioimaging [69–97]. Since the chemical shift depends on vector *L* between the proton and the unpaired electrons, factors like temperature and protonation can alter the geometry and thus change the relative shift. The total shift term, Δ*δ*_*O*_, is modeled as

(4) $$\begin{array}{c} \Delta \delta_{O}=C_{T}\Delta T+C_{pH}\Delta pH+C_{X}\Delta \left[\;X\;\right], \end{array}$$

where both temperature (*T*) and pH can change simultaneously, *C*_*T*_ = (Δ*δ*_*O*_/Δ*T*)_pH_ is the temperature dependence at a given pH, *C*_pH_ = (Δ*δ*_*O*_/ΔpH)_*T*_ is the pH dependence at a given temperature, and the much weaker *C*_*X*_ term is for effects arising from cation *X*. Because the molecular readout by BIRDS is based on shifts, the method is independent of agent dose, diffusion, blood flow, and vessel permeability. However ROIs with high vessel permeability (e.g., tumor tissue) reveal higher peaks.

Recent BIRDS studies by Coman and coworkers [41] and Huang and coworkers [20], both at *B*_*o*_ of 11.7 T using a pH-sensitive TmDOTP^5−^ probe, measured pH_e_ in rat brain containing various types of gliomas, that is, 9L, RG2, and U87 [20, 41]. The agent's clearance was perturbed to build up the agent concentration in the circulation, either by injecting the agent alone upon renal ligation [41] or by coinjecting the agent with probenecid (i.e., an organic anion transporter inhibitor) to enable longitudinal scans [20]. Generally a higher agent concentration and thus higher SNR for BIRDS was achieved with the former method, but the pH_e_ mapping was shown to be independent of the agent dose using the latter method.

**Figure 6:**
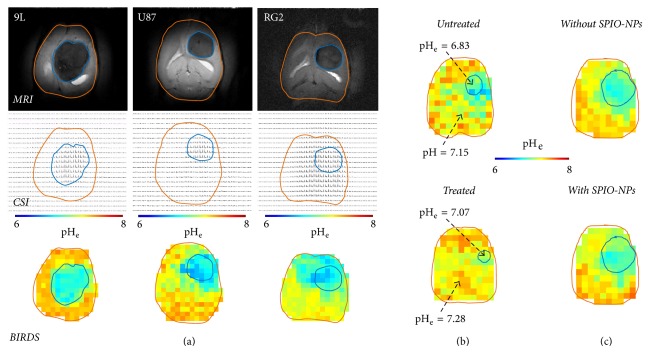
BIRDS-based pH_e_ mapping of different brain tumors at *B*_*o*_ of 11.7 T. (a) Representative *T*_2_-weighted MRI with TmDOTP^5−^ (top row), CSI of protons on TmDOTP^5−^ (middle row), and pH_e_ map by BIRDS (bottom row) of rats bearing 9L, U87, and RG2 tumors during probenecid-TmDOTP^5−^ coinfusion. The tumor boundaries in CSI and BIRDS data (blue line) are from the MRI-defined region outlined by contrast enhancement arising from TmDOTP^5−^. From [20] with permission. (b) Representative pH_e_ maps from BIRDS in untreated (top) and temozolomide-treated (bottom) different rats bearing U251 tumors. The tumor boundary in BIRDS data (blue line) are from the MRI-defined region outlined by contrast enhancement arising from TmDOTP^5−^. From [21] with permission. (c) Representative pH_e_ maps from BIRDS before (top) and after (bottom) injection of SPIO-NPs in the same rat bearing an RG2 tumor. A SPIO-NPs dose of 7.3 mg/kg Fe^3+^ was injected systemically. The tumor boundaries in BIRDS data (blue line) are from the MRI-defined region outlined by contrast enhancement arising from SPIO-NPs dose of 1.7 mg/kg Fe^3+^ in the tumor. From [22] with permission.

Upon BIRDS agent infusion, MRI identified the tumor boundary by enhanced relaxation because of TmDOTP^5−^ (i.e., its paramagnetic effect on *R*_2_ of water protons) and BIRDS allowed pH_e_ mapping of brains with different gliomas (Figure 6(a)). While the intratumoral pH_e_ was acidic for all glioma types, the peritumoral pH_e_ varied with the tumor type. For example, in RG2 (and U87) tumors acidic pH_e_ was found in distal peritumoral regions beyond the RG2 (and U87) tumor border which corresponded to increased presence of Ki-67 positive cells, and this was not the case with 9L tumor which is a far less aggressive tumor [41].

Given these exciting pH_e_ results with BIRDS, Rao and coworkers measured U251 tumors with and without temozolomide, a DNA alkylating agent [21]. Treated rats had reduced tumor volume and higher pH_e_ compared to untreated rats (Figure 6(b)), and these findings were supported by reduced proliferation (Ki-67 staining) and apoptosis induction (cleaved caspase-3 staining) examined within the tumor boundaries of treated rats. Since GBM treatment is hampered by a lack of bioimaging methodologies that can simultaneously and noninvasively measure location of F-NPs and response to therapeutic benefits of F-NPs, Maritim and coworkers [22] demonstrated that quantitative pH_e_ imaging using BIRDS in different gliomas is compatible with MRI contrast from SPIO-NPs for tumor delineation. It was found that the pH_e_ inside and outside the MRI-defined tumor boundary remained unaffected after the infusion of 7.3 mg/kg Fe^3+^ SPIO-NPs (Figure 6(c)), regardless of whether the tumor was aggressive or not (9L versus RG2) or the agent injection method was terminal or not (renal ligation versus coinfusion with probenecid).

## 5. Future Theranostics for Brain Tumors

**Figure 7:**
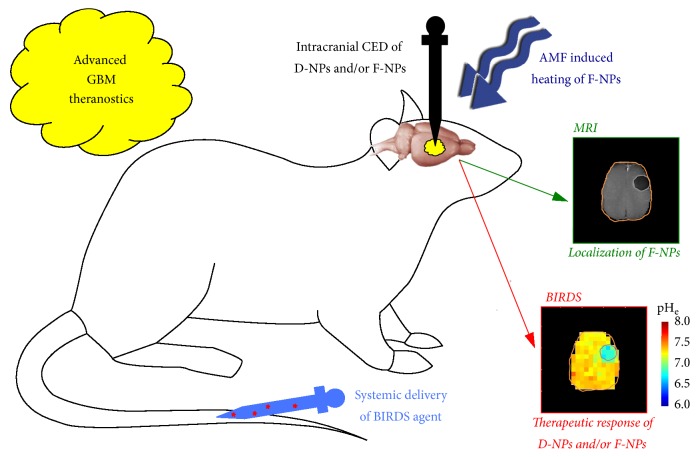
Advanced GBM theranostics. Intracranial CED of D-NPs is used to deliver high concentration chemotherapy over prolonged periods directly into the tumor. However when intracranial CED of D-NPs is combined with F-NPs, MRI can be used to monitor the location of the chemotherapy. Alternatively, intracranial CED of F-NPs alone can be used to provide thermotherapy by AMF-induced heating. BIRDS, which requires systemic injection of an imaging agent, can measure responses to chemotherapy from D-NPs and thermotherapy from F-NPs, because BIRDS is unaffected by the superparamagnetic field gradients generated by F-NPs. The MRI and BIRDS data shown are from [22] with permission.

The aforementioned advances show great potential for GBM theranostics with current technologies to impact* both* chemotherapy from D-NPs and thermotherapy from F-NPs. The therapeutic response can be measured by BIRDS, and this can be achieved at the same time as determining the location of F-NPs by MRI (Figure 7). But new imaging methods could provide greater metabolic insights. Unlike BIRDS, which allows pH_e_ mapping, CEST-based imaging of pH_i_ is incompatible with F-NPs at doses greater than 5 mg/kg Fe^3+^ because superparamagnetic field gradients create challenges for MRI contrast (due to extensive spatial distortions) [47] and MRS specificity (due to extreme line broadening) [48] needed for molecular imaging. Although in vivo pH_e_ mapping by BIRDS has been shown to be unaffected with SPIO-NPs dose of ~7 mg/kg Fe^3+^ [22], in vitro studies suggest that the pH_e_ mapping limit can extend up to 3 times higher doses of SPIO-NPs [68]. However, whenever D-NPs alone are used, CEST and BIRDS can be combined to obtain* both *pH_i_ and pH_e_ information together. While FDA-approved versions of F-NPs are already available, further research and approvals are needed to translate novel imaging methods that require infusion of exogenous imaging probes. Regardless of this, these novel imaging methods are valuable for preclinical research and have significant clinical relevance because xenografted human GBM cells directly injected into an animal brain represent an accurate GBM model [98–100].

Nanoparticles can be bioengineered for drug delivery into the brain [101] using endogenous methods like transcytosis (e.g., adsorptive-mediated [102], carrier-mediated [103], and receptor-mediated [104]) or targeting the disease itself [105] to transient disruption of the BBB by ultrasound [106]. Future designs of F-NPs (or D-NPs) could incorporate these modifications to their surfaces for improved cancer targeting, which may even alleviate the need for intraoperative CED. However these surface modifications of F-NPs should be attentive to anisotropy, hydrodynamic volume, and physical size, which all greatly influence the effective relaxation enhancement. Since F-NPs are not colloidally stable in aqueous media, the biocompatible coatings should allow for some electrostatic charge build-up. The imaging advances in conjunction with smarter designs of agents could be even greater for GBM theranostics. Compatibility of BIRDS with various nanoensembles such as SPIO-NPs, GdDTPA^2−^, liposomes, and even dendrimers [68, 107] may create exciting opportunities for multimodal imaging of drug response. Therefore, development of next generation imaging agents along with surface modification of nanoparticles will allow simultaneous detection of therapy response and location will greatly impact GBM theranostics.

## Figures and Tables

**Table 1:** Physical characterization of Fe_*x*_Co_1__−__*x*_Fe_2_O_4_ (where *x* = 0.8,0.6,0.4,0.2) for bare and coated (chitosan, PEG) nanoparticles using different coprecipitating agents (NH_4_OH, NaOH) for chemical synthesis. See Figure 4 for other details [10].

*x*	Method used	Grain size (nm)	Lattice (Å)	Blocking temperature (°C)	Hydrodynamic diameter (nm)		Maximum magnetization (emu/g)			Coercivity (Oe)			Relaxivity (mM^−1^s^−1^)	
					Chitosan	PEG	Bare	Chitosan	PEG	Bare	Chitosan	PEG	Chitosan	PEG
0.8	NH_4_OH	4.0 (±0.6)	8.48 (±0.06)	−100	97.5	158.3	59.3	37.2	26.0	3.5	2.08	1.4	528 (±26)	314 (±25)
0.6	NH_4_OH	4.5 (±0.1)	8.52 (±0.26)	25	135.5	154.5	55.3	28.0	20	20	7.5	8.6	509 (±45)	441 (±12)
0.4	NH_4_OH	2.8 (±0.1)	8.45 (±0.20)	65	140.6	159.1	43.6	25.2	16.9	157	4.2	4.8	326 (±37)	310 (±5)
0.2	NH_4_OH	—	—	100	110.0	155.5	32.3	—	—	506	—	—	—	—
0.2	NaOH	4.9 (±0.1)	8.43 (±0.05)	—	—	—	73.1	54.5	46.6	673	11.5	7.8	769 (±92)	377 (±14)

## Abbreviations

AACID: Amine and amide concentration-independent detection
AMF: Alternating magnetic field
3-APP: 3-Aminopropyl phosphonate
APT: Amide proton transfer
*B*_*o*_: Static magnetic field
BBB: Blood-brain barrier
BIRDS: Biosensor imaging of redundant deviation in shifts
CED: Convection-enhanced delivery
CEST: Chemical exchange saturation transfer
CSI: Chemical shift imaging
DOTA^4−^: 1,4,7,10-Tetraazacyclododecane-1,4,7,10-tetraacetic acid
DOTMA^4−^: 1,4,7,10-Tetramethyl 1,4,7,10-tetraazacyclododecane-1,4,7,10-tetraacetate
DOTP^8−^: 1,4,7,10-Tetraazacyclododecane-1,4,7,10-tetrakis methylene phosphonate
DTPA^5−^: Diethylenetriamine pentaacetic acid
D-NPs: Drug-containing nanoparticles
F-NPs: Ferrite nanoparticles
FDA: Federal Drug Agency
GBM: Glioblastoma multiforme
M-NPs: Magnetic nanoparticles
MRI: Magnetic resonance imaging
MRS: Magnetic resonance spectroscopy
pH_e_: Extracellular pH
pH_i_: Intracellular pH
P_i_: Inorganic phosphate
RF: Radio frequency
ROI: Region of interest
SLP: Specific loss power
SNR: Signal-to-noise ratio
SPIO-NPs: Superparamagnetic iron oxide nanoparticles
TEM: Transmission electron microscopy
XRD: X-ray diffraction.

## References

[1] Asthagiri A. R., Pouratian N., Sherman J., Ahmed G., Shaffrey M. E. (2007). Advances in Brain Tumor Surgery. *Neurologic Clinics*.

[2] http://www.cancer.gov/, 2014

[3] Scott C. B., Scarantino C., Urtasun R. Validation and predictive power of Radiation Therapy Oncology Group (RTOG) recursive partitioning analysis classes for malignant glioma patients: a report using RTOG 90-06. *International Journal of Radiation Oncology, Biology, Physics*.

[4] Zhou J., Atsina K.-B., Himes B. T., Strohbehn G. W., Saltzman W. M. (2012). Novel delivery strategies for glioblastoma. *Cancer Journal*.

[5] Sathornsumetee S., Rich J. N. (2006). New treatment strategies for malignant gliomas. *Expert Review of Anticancer Therapy*.

[6] Pardridge W. M. (2007). Blood-brain barrier delivery. *Drug Discovery Therapy*.

[7] Blakeley J. (2008). Drug delivery to brain tumors. *Current Neurology and Neuroscience Reports*.

[8] Gutenberg A., Lumenta C. B., Braunsdorf W. E. K. (2013). The combination of carmustine wafers and temozolomide for the treatment of malignant gliomas. a comprehensive review of the rationale and clinical experience. *Journal of Neuro-Oncology*.

[9] Re F., Gregori M., Masserini M. (2012). Nanotechnology for neurodegenerative disorders. *Nanomedicine: Nanotechnology, Biology and Medicine*.

[10] Hoque S. M., Huang Y., Cocco E. (2016). Improved specific loss power on cancer cells by hyperthermia and MRI contrast of hydrophilic FexCo1-xFe2O4 nanoensembles. *Contrast Media & Molecular Imaging*.

[11] Modi G., Pillay V., Choonara Y. E., Ndesendo V. M. K., du Toit L. C., Naidoo D. (2009). Nanotechnological applications for the treatment of neurodegenerative disorders. *Progress in Neurobiology*.

[12] Zhou J., Patel T. R., Sirianni R. W. (2013). Highly penetrative, drug-loaded nanocarriers improve treatment of glioblastoma. *Proceedings of the National Acadamy of Sciences of the United States of America*.

[13] Wang A. Z., Langer R., Farokhzad O. C. (2012). Nanoparticle delivery of cancer drugs. *Annual Review of Medicine*.

[14] Kefeni K. K., Msagati T. A. M., Mamba B. B. (2017). Ferrite nanoparticles: Synthesis, characterisation and applications in electronic device. *Materials Science and Engineering: B Advanced Functional Solid-State Materials*.

[15] Gatenby R. A., Gillies R. J. (2004). Why do cancers have high aerobic glycolysis?. *Nature Reviews Cancer*.

[16] Hashim A. I., Zhang X., Wojtkowiak J. W., Martinez G. V., Gillies R. J. (2011). Imaging pH and metastasis. *NMR in Biomedicine*.

[17] Allen S. J., Watson J. J., Shoemark D. K., Barua N. U., Patel N. K. (2013). GDNF, NGF and BDNF as therapeutic options for neurodegeneration. *Pharmacology & Therapeutics*.

[18] Mardor Y., Rahav O., Zauberman Y. (2005). Convection-enhanced drug delivery: Increased efficacy and magnetic resonance image monitoring. *Cancer Research*.

[19] Strohbehn G., Coman D., Han L. (2015). Imaging the delivery of brain-penetrating PLGA nanoparticles in the brain using magnetic resonance. *Journal of Neuro-Oncology*.

[20] Huang Y., Coman D., Herman P., Rao J. U., Maritim S., Hyder F. (2016). Towards longitudinal mapping of extracellular pH in gliomas. *NMR in Biomedicine*.

[21] Rao J. U., Coman D., Walsh J. J., Ali M. M., Huang Y., Hyder F. (2017). Temozolomide arrests glioma growth and normalizes intratumoral extracellular pH. *Scientific Reports*.

[22] Maritim S., Coman D., Huang Y., Rao J. U., Walsh J. J., Hyder F. Mapping extracellular pH of gliomas in presence of superparamagnetic nanoparticles: towards imaging the distribution of drug-containing nanoparticles and their curative effect on the tumor microenvironment. *Contrast Media and Molecular Imaging*.

[23] Hunt Bobo R., Laske D. W., Akbasak A., Morrison P. F., Dedrick R. L., Oldfield E. H. (1994). Convection-enhanced delivery of macromolecules in the brain. *Proceedings of the National Acadamy of Sciences of the United States of America*.

[24] Kunwar S., Chang S., Westphal M. (2010). Phase III randomized trial of CED of IL13-PE38QQR vs Gliadel wafers for recurrent glioblastoma. *Neuro-Oncology*.

[25] Sampson J. H., Archer G., Pedain C. (2010). Poor drug distribution as a possible explanation for the results of the PRECISE trial. *Journal of Neurosurgery*.

[26] Sarin H. (2010). On the future development of optimally-sized lipid-insoluble systemic therapies for CNS solid tumors and other neuropathologies. *Recent Patents on CNS Drug Discovery*.

[27] Pankhurst Q. A., Connolly J., Jones S. K., Dobson J. (2003). Applications of magnetic nanoparticles in biomedicine. *Journal of Physics D: Applied Physics*.

[28] Lee J., Huh Y., Jun Y. (2007). Artificially engineered magnetic nanoparticles for ultra-sensitive molecular imaging. *Nature Medicine*.

[29] Thu M. S., Bryant L. H., Coppola T. (2012). Self-assembling nanocomplexes by combining ferumoxytol, heparin and protamine for cell tracking by magnetic resonance imaging. *Nature Medicine*.

[30] Okuhata Y. (1999). Delivery of diagnostic agents for magnetic resonance imaging. *Advanced Drug Delivery Reviews*.

[31] Na H. B., Song I. C., Hyeon T. (2009). Inorganic nanoparticles for MRI contrast agents. *Advanced Materials*.

[32] Ghosh R., Pradhan L., Devi Y. P. (2011). Induction heating studies of Fe3O4 magnetic nanoparticles capped with oleic acid and polyethylene glycol for hyperthermia. *Journal of Materials Chemistry*.

[33] Ward K. M., Balaban R. S. (2000). Determination of pH using water protons and chemical exchange dependent saturation transfer (CEST). *Magnetic Resonance in Medicine*.

[34] Sagiyama K., Mashimo T., Togao O. (2014). In vivo chemical exchange saturation transfer imaging allows early detection of a therapeutic response in glioblastoma. *Proceedings of the National Acadamy of Sciences of the United States of America*.

[35] McVicar N., Li A. X., Gonçalves D. F. (2014). Quantitative tissue pH measurement during cerebral ischemia using amine and amide concentration-independent detection (AACID) with MRI. *Journal of Cerebral Blood Flow & Metabolism*.

[36] Gillies R. J., Liu Z., Bhujwalla Z. (1994). 31P-MRS measurements of extracellular pH of tumors using 3- aminopropylphosphonate. *American Journal of Physiology-Cell Physiology*.

[37] Chen L. Q., Randtke E. A., Jones K. M., Moon B. F., Howison C. M., Pagel M. D. (2015). Evaluations of tumor acidosis within in vivo tumor models using parametric maps generated with AcidoCEST MRI. *Molecular Imaging and Biology*.

[38] Yoo B., Pagel M. D. (2008). An overview of responsive MRI contrast agents for molecular imaging. *Frontiers in Bioscience*.

[39] Coman D., Trubel H. K., Rycyna R. E., Hyder F. (2009). Brain temperature and pH measured by ^1^H chemical shift imaging of a thulium agent. *NMR in Biomedicine*.

[40] Coman D., De Graaf R. A., Rothman D. L., Hyder F. (2013). In vivo three-dimensional molecular imaging with Biosensor Imaging of Redundant Deviation in Shifts (BIRDS) at high spatiotemporal resolution. *NMR in Biomedicine*.

[41] Coman D., Huang Y., Rao J. U. (2016). Imaging the intratumoral-peritumoral extracellular pH gradient of gliomas. *NMR in Biomedicine*.

[42] Hyder F., Rothman D. L. (2017). Advances in Imaging Brain Metabolism. *Annual Review of Biomedical Engineering*.

[43] Warburg O. (1956). On the origin of cancer cells. *Science*.

[44] Harris R. J., Cloughesy T. F., Liau L. M. (2015). pH-weighted molecular imaging of gliomas using amine chemical exchange saturation transfer MRI. *Neuro-Oncology*.

[45] Bhujwalla Z. M., Artemov D., Ballesteros P., Cerdan S., Gillies R. J., Solaiyappan M. (2002). Combined vascular and extracellular pH imaging of solid tumors. *NMR in Biomedicine*.

[46] Raghunand N., Altbach M. I., Van Sluis R. (1999). Plasmalemmal pH-gradients in drug-sensitive and drug-resistant MCF-7 human breast carcinoma xenografts measured by 31P magnetic resonance spectroscopy. *Biochemical Pharmacology*.

[47] Murillo T. P., Sandquist C., Jacobs P. M., Nesbit G., Manninger S., Neuwelt E. A. (2005). Imaging brain tumors with ferumoxtran-10, a nanoparticle magnetic resonance contrast agent. *Thérapie*.

[48] Le Duc G., Elst L. V., Colet J. M. (2001). Ultrasmall particulate iron oxides as contrast agents for magnetic resonance spectroscopy: A dose-effect study. *Journal of Magnetic Resonance Imaging*.

[49] Gilchrist R. K., Medal R., Shorey W. D., Hanselman R. C., Parrott J. C., Taylor C. B. (1957). Selective inductive heating of lymph nodes. *Annals of Surgery*.

[50] Lee J.-H., Jang J.-T., Choi J.-S. (2011). Exchange-coupled magnetic nanoparticles for efficient heat induction. *Nature Nanotechnology*.

[51] Issa B., Obaidat I. M., Albiss B. A., Haik Y. (2013). Magnetic nanoparticles: Surface effects and properties related to biomedicine applications. *International Journal of Molecular Sciences*.

[52] Roch A., Muller R. N., Gillis P. (1999). Theory of proton relaxation induced by superparamagnetic particles. *The Journal of Chemical Physics*.

[53] Joshi H. M., Lin Y. P., Aslam M. (2009). Effects of shape and size of cobalt ferrite nanostructures on their MRI contrast and thermal activation. *The Journal of Physical Chemistry C*.

[54] Duan X., Li Y. (2013). Physicochemical characteristics of nanoparticles affect circulation, biodistribution, cellular internalization, and trafficking. *Small*.

[55] Nándori I., Rácz J. (2012). Magnetic particle hyperthermia: Power losses under circularly polarized field in anisotropic nanoparticles. *Physical Review E: Statistical, Nonlinear, and Soft Matter Physics*.

[56] Fortin J.-P., Wilhelm C., Servais J., Ménager C., Bacri J.-C., Gazeau F. (2007). Size-sorted anionic iron oxide nanomagnets as colloidal mediators for magnetic hyperthermia. *Journal of the American Chemical Society*.

[57] Prado Y., Daffé N., Michel A. (2015). Enhancing the magnetic anisotropy of maghemite nanoparticles via the surface coordination of molecular complexes. *Nature Communications*.

[58] Poperechny I. S., Raikher Y. L., Stepanov V. I. (2010). Dynamic magnetic hysteresis in single-domain particles with uniaxial anisotropy. *Physical Review B: Condensed Matter and Materials Physics*.

[59] Habib A. H., Ondeck C. L., Chaudhary P., Bockstaller M. R., McHenry M. E. (2008). Evaluation of iron-cobalt/ferrite core-shell nanoparticles for cancer thermotherapy. *Journal of Applied Physics*.

[60] Tackett R., Sudakar C., Naik R., Lawes G., Rablau C., Vaishnava P. P. (2008). Magnetic and optical response of tuning the magnetocrystalline anisotropy in Fe3O4 nanoparticle ferrofluids by Co doping. *Journal of Magnetism and Magnetic Materials*.

[61] Hoque S. M., Hossain M. S., Choudhury S., Akhter S., Hyder F. (2016). Synthesis and characterization of ZnFe2O4 nanoparticles and its biomedical applications. *Materials Letters*.

[62] Palchoudhury S., Hyder F., Vanderlick T. K., Geerts N. (2014). Water-soluble anisotropic iron oxide nanoparticles: Dextran-coated crystalline nanoplates and nanoflowers. *Particulate Science and Technology*.

[63] Singh N., Jenkins G. J., Asadi R., Doak S. H. (2010). Potential toxicity of superparamagnetic iron oxide nanoparticles (SPION). *Nano Reviews*.

[64] Szerlip N. J., Walbridge S., Yang L. (2007). Real-time imaging of convection-enhanced delivery of viruses and virus-sized particles. *Journal of Neurosurgery*.

[65] Coman D., Trubel H. K., Hyder F. (2010). Brain temperature by Biosensor Imaging of Redundant Deviation in Shifts (BIRDS): Comparison between TmDOTP5- and TmDOTMA-. *NMR in Biomedicine*.

[66] Abragam A. (1961). *Principles of Nuclear Magnetism*.

[67] Trübel H. K. F., Maciejewski P. K., Farber J. H., Hyder F. (2003). Brain temperature measured by 1H-NMR in conjunction with a lanthanide complex. *Journal of Applied Physiology*.

[68] Maritim S., Huang Y., Coman D., Hyder F. (2014). Characterization of a lanthanide complex encapsulated with MRI contrast agents into liposomes for biosensor imaging of redundant deviation in shifts (BIRDS). *Journal of Biological Inorganic Chemistry*.

[69] Buster D. C., Castro M. M. C. A., Geraldes C. F. G. C., Malloy C. R., Sherry A. D., Siemers T. C. (1990). Tm(DOTP)5−: A 23Na+ shift agent for perfused rat hearts. *Magnetic Resonance in Medicine*.

[70] Bansal N., Germann M. J., Lazar I., Malloy C. R., Sherry A. D. (1992). In vivo Na‐23 MR imaging and spectroscopy of rat brain during TmDOTP5− infusion. *Journal of Magnetic Resonance Imaging*.

[71] Bansal N., Germann M. J., Seshan V., Shires G. T., Malloy C. R., Sherry A. D. (1993). Thulium 1,4,7,10-tetraazacyclododecane-1,4,7,10-tetrakis(methylene phosphonate) as a 23Na shift reagent for the in vivo rat liver. *Biochemistry*.

[72] Geraldes C. F. G. C., Sherry A. D., Lázár I. (1993). Relaxometry, animal biodistribution, and magnetic resonance imaging studies of some new gadolinium (III) macrocyclic phosphinate and phosphonate monoester complexes. *Magnetic Resonance in Medicine*.

[73] Xia Z. F., Horton J. W., Zhao P. Y. (1994). In vivo studies of cellular energy state, pH, and sodium in rat liver after thermal injury. *J Appl Physiol*.

[74] Seshan V., Germann M. J., Preisig P., Malloy C. R., Dean Sherry A., Bansal N. (1995). Tmdotp5− as a 23na shift reagent for the in vivo rat kidney. *Magnetic Resonance in Medicine*.

[75] Sherry A. D., Ren J., Huskens J. (1996). Characterization of Lanthanide(III) DOTP complexes: thermodynamics, protonation, and coordination to alkali metal ions. *Inorganic Chemistry*.

[76] Zuo C. S., Bowers J. L., Metz K. R., Nosaka T., Sherry A. D., Clouse M. E. (1996). TmDOTP5-: A substance for NMR temperature measurements in vivo. *Magnetic Resonance in Medicine*.

[77] Kim W. D., Kiefer G. E., Huskens J., Dean Sherry A. (1997). NMR Studies of the Lanthanide(III) Complexes of 1,4,7,10-Tetraazacyclododecane-l,4,7,10-tetrakis(methanephosphonic acid mono(2',2',2'-trifluoroethyl) ester). *Inorganic Chemistry*.

[78] Winter P. M., Seshan V., Makos J. D., Sherry A. D., Malloy C. R., Bansal N. (1998). Quantitation of intracellular [Na+] in vivo by using TmDOTP5- as an NMR shift reagent and extracellular marker. *Journal of Applied Physiology*.

[79] Zuo C. S., Metz K. R., Sun Y., Sherry A. D. (1998). NMR temperature measurements using a paramagnetic lanthanide complex. *Journal of Magnetic Resonance*.

[80] Colet J.-M., Bansal N., Malloy C. R., Sherry A. D. (1999). Multiple quantum filtered 23Na NMR spectroscopy of the isolated, perfused rat liver. *Magnetic Resonance in Medicine*.

[81] Winter P. M., Bansal N. (2001). Triple-quantum-filtered (23)Na NMR spectroscopy of subcutaneously implanted 9l gliosarcoma in the rat in the presence of TmDOTP(5-1). *Journal of Magnetic Resonance*.

[82] Winter P. M., Bansal N. (2001). TmDOTP^5-^ as a ^23^Na shift reagent for the subcutaneously implanted 9L gliosarcoma in rats. *Magnetic Resonance in Medicine*.

[83] Zhou R., Bansal N., Leeper D. B., Pickup S., Glickson J. D. (2001). Enhancement of hyperglycemia-induced acidification of human melanoma xenografts with inhibitors of respiration and ion transport. *Academic Radiology*.

[84] Hekmatyar S. K., Poptani H., Babsky A., Leeper D. B., Bansal N. (2002). Non-invasive magnetic resonance thermometry using thulium-1,4,7,10-tetraazacyclododecane-1,4,7,10-tetraacetate (TmDOTA(-)). *International Journal of Hyperthermia*.

[85] Babsky A., Hekmatyar S. K., Wehrli S., Nelson D., Bansal N. (2004). Effects of temperature on intracellular sodium, pH and cellular energy status in RIF-1 tumor cells. *NMR in Biomedicine*.

[86] Babsky A., Hekmatyar S. K., Gorski T., Nelson D. S., Bansal N. (2005). Heat-induced changes in intracellular Na+, pH and bioenergetic status in superfused RIF-1 tumour cells determined by 23Na and 31P magnetic resonance spectroscopy. *International Journal of Hyperthermia*.

[87] Babsky A. M., Hekmatyar S. K., Zhang H., Solomon J. L., Bansal N. (2005). Application of23Na MRI to monitor chemotherapeutic response in RIF-1 tumors. *Neoplasia*.

[88] Hekmatyar S. K., Hopewell P., Pakin S. K., Babsky A., Bansal N. (2005). Noninvasive MR thermometry using paramagnetic lanthanide complexes of 1,4,7,10-tetraazacyclodoecane-alpha, alpha', alpha'', alpha'''-tetramethyl-1, 4,7,10-tetraacetic acid (DOTMA4-). *Magnetic Resonance in Medicine*.

[89] Hekmatyar S. K., Kerkhoff R. M., Pakin S. K., Hopewell P., Bansal N. (2005). Noninvasive thermometry using hyperfine-shifted MR signals from paramagnetic lanthanide complexes. *International Journal of Hyperthermia*.

[90] Pakin S. K., Hekmatyar S. K., Hopewell P., Babsky A., Bansal N. (2006). Non-invasive temperature imaging with thulium 1,4,7, 10-tetraazacyclododecane- 1,4,7, 10-tetramethyl-1,4,7, 10-tetraacetic acid (TmDOTMA-). *NMR in Biomedicine*.

[91] Hentschel M., Wust P., Wlodarczyk W. (1998). Non-invasive MR thermometry by 2D spectroscopic imaging of the Pr[MOE-DO3A] complex. *International Journal of Hyperthermia*.

[92] Wlodarczyk W., Boroschewski R., Hentschel M., Wust P., Mönich G., Felix R. (1998). Three-dimensional monitoring of small temperature changes for therapeutic hyperthermia using MR. *Journal of Magnetic Resonance Imaging*.

[93] Hentschel M., Dreher W., Wust P., Röll S., Leibfritz D., Felix R. (1999). Fast spectroscopic imaging for non-invasive thermometry using the Pr[MOE-DO3A] complex. *Physics in Medicine and Biology*.

[94] Wlodarczyk W., Hentschel M., Wust P. (1999). Comparison of four magnetic resonance methods for mapping small temperature changes. *Physics in Medicine and Biology*.

[95] Chalmers K. H., De Luca E., Hogg N. H. M. (2010). Design principles and theory of paramagnetic fluorine-labelled lanthanide complexes as probes for (19)F magnetic resonance: a proof-of-concept study. *Chemistry - A European Journal*.

[96] Senanayake P. K., Rogers N. J., Finney K.-L. N. A. (2017). A new paramagnetically shifted imaging probe for MRI. *Magnetic Resonance in Medicine*.

[97] Finney K. N., Harnden A. C., Rogers N. J. (2017). Simultaneous Triple Imaging with Two PARASHIFT Probes: Encoding Anatomical, pH and Temperature Information using Magnetic Resonance Shift Imaging. *Chemistry - A European Journal*.

[98] Hulsey K. M., Mashimo T., Banerjee A. (2015). (1)H MRS characterization of neurochemical profiles in orthotopic mouse models of human brain tumors. *NMR in Biomedicine*.

[99] Ozawa T., James C. D. (2010). Establishing intracranial brain tumor xenografts with subsequent analysis of tumor growth and response to therapy using bioluminescence imaging.. *Journal of visualized experiments : JoVE*.

[100] El Meskini R., Iacovelli A. J., Kulaga A. (2015). A preclinical orthotopic model for glioblastoma recapitulates key features of human tumors and demonstrates sensitivity to a combination of MEK and PI3K pathway inhibitors. *Disease Models & Mechanisms*.

[101] Deeken J. F., Löscher W. (2007). The blood-brain barrier and cancer: transporters, treatment, and trojan horses. *Clinical Cancer Research*.

[102] Liu L., Venkatraman S. S., Yang Y.-Y. (2008). Polymeric micelles anchored with TAT for delivery of antibiotics across the blood-brain barrier. *Biopolymers - Peptide Science Section*.

[103] Li J., Guo Y., Kuang Y., An S., Ma H., Jiang C. (2013). Choline transporter-targeting and co-delivery system for glioma therapy. *Biomaterials*.

[104] Qiao R., Jia Q., Hüwel S. (2012). Receptor-mediated delivery of magnetic nanoparticles across the blood-brain barrier. *ACS Nano*.

[105] Kievit F. M., Veiseh O., Fang C. (2010). Chlorotoxin labeled magnetic nanovectors for targeted gene delivery to glioma. *ACS Nano*.

[106] Nance E., Timbie K., Miller G. W. (2014). Non-invasive delivery of stealth, brain-penetrating nanoparticles across the blood - Brain barrier using MRI-guided focused ultrasound. *Journal of Controlled Release*.

[107] Huang Y., Coman D., Hyder F., Ali M. M. (2015). Dendrimer-Based Responsive MRI Contrast Agents (G1-G4) for Biosensor Imaging of Redundant Deviation in Shifts (BIRDS). *Bioconjugate Chemistry*.

